# Identification and validation of necroptosis-related genes in peripheral blood mononuclear cells of Sjögren disease

**DOI:** 10.3389/fmed.2026.1727374

**Published:** 2026-03-26

**Authors:** Yuanji Dong, Lingli Dong, Rongfen Gao

**Affiliations:** 1 Department of Rheumatology and Immunology, Tongji Hospital, Tongji Medical College, Huazhong University of Science and Technology, Wuhan, Hubei, China; 2 Department of Rheumatology, The Second Affiliated Hospital of Zhejiang University School of Medicine, Hangzhou, Zhejiang, China; 3 Department of Rheumatology and Immunology, Tongji Medical College and State Key Laboratory for Diagnosis and Treatment of Severe Zoonotic Infectious Disease, Huazhong University of Science and Technology, Wuhan, Hubei, China

**Keywords:** HMGB1, necroptosis, PBMCs, Sjögren disease, therapeutic target

## Abstract

**Background:**

Necroptosis has been implicated in multiple inflammatory and autoimmune disorders. However, its role in Sjögren disease (SjD) remains elucidated. In the current study, we aimed to identify and validate SjD-associated necroptosis genes.

**Methods:**

Differentially expressed genes were analyzed with GSE48378 downloaded from the GEO database using the DESeq2 R package. GO enrichment analysis and KEGG pathway enrichment analysis were carried out. Then, the necroptosis-relevant genes were subjected to PPI analysis. RT-PCR was used to verify the expression of the top 8 and the key 2 necroptosis genes, as well as HMGB1 expression. ELISA detected the concentration of HMGB1. The correlations of MLKL or HMGB1 with clinical parameters of SjD were also evaluated. The electron microscope confirmed whether necroptosis had occurred or not.

**Results:**

Twenty-four necroptosis-related genes were found in the PBMCs from SjD patients, all closely interacting as determined by PPI analysis. We established a validation cohort and selected the top eight differential necroptosis genes and two critical necroptosis genes from 24. We found that 7 of 10 (TNFRSF10, STAT3, NLRP3, RIPK, MLKL, TLR4, and EIF2AK2) were elevated in SjD, consistent with the bioinformatics analysis. We also found that HMGB1 levels in the peripheral blood of SjD were elevated and positively correlated with ESSDAI. Meanwhile, we found that the morphological features of cell death observed in PBMCs were consistent with those described for necroptosis in the PBMC of the SjD.

**Conclusion:**

The bioinformatics analysis identified an enrichment of the necroptosis pathway in the PBMCs of SjD, which was validated in a real-world cohort. Meanwhile, we found that HMGB1 levels were increased in peripheral blood and hypothesize that this elevation may be partly attributable to pyroptosis, despite the current lack of more substantial evidence. HMGB1 is also positively correlated with ESSDAI, suggesting that it may represent a novel therapeutic target.

## Introduction

Sjögren disease (SjD) is a common systemic autoimmune disease (1). The clinical presentation can vary greatly, ranging from the asymptomatic, fatigue or pain, and predominant gland involvement to extra-glandular manifestations (including skin, joints, muscles, nervous system, lungs, and kidneys), and even lymphoma (2). It is reported that the incidence of SjD is estimated to be between 1–10 per 10^5^ PY (person-year), with multiple estimates of around 5 per 10^5^ PY (3). Among these patients, over 50% experience extra-glandular manifestations, and approximately 2.7 to 9.8% develop lymphoma (4, 5). With the growing knowledge of the pathogenesis of SjD, particularly in terms of the interaction between epithelial cells and immune cells, it has been discovered that epithelial cells can express co-stimulatory molecules and interact with T cells to promote their differentiation into helper T cell 1 (Th1) and follicular helper T (Tfh) subsets (6, 7). Epithelial cells are also capable of secreting various cytokines, such as the B cell activation factor of the tumor necrosis factor family (BAFF) and IL-6, which are involved in B cell activation and homeostasis (8). In turn, B cells can induce transcriptional changes in epithelial cells, promoting the development of lymphoepithelial lesions (LELs), which can lead to obstruction of salivary ducts and decreased salivary secretion (9). In addition, the plasmacytoid dendritic cells (pDC)/type 1 interferon axis, IL-23/IL-17 axis, as well as tissue-infiltrating macrophages, tissue-resident CD103-negative CD8^+^ T cells, and CD11c^+^ T-bet^+^ FcRL4^+^ B cells have all been involved in the pathogenesis of SjD, which broadens our understanding of molecular regulation of SjD (9–13).

The treatment options for SjD remain rather limited. Traditional treatment methods include topical administration of artificial tears or saliva and systemic administration of glucocorticoids or immunosuppressants (14). It is heartening that several new approaches have been attempted in SjD, such as targeting interferon, targeting BAFF receptors, targeting co-stimulatory molecules, or targeting Janus kinase (JAK), etc. (15). However, compared to other systemic autoimmune diseases, breakthrough treatment for SjD is still lacking (16). Necroptosis is a programmed form of necrosis triggered by various stimuli, such as DNA damage or infection. Compared to the traditional non-inflammatory apoptosis, necroptosis has a strong pro-inflammatory effect (17). It is reported that necroptosis can lead to the release of damage-associated molecular patterns (DAMPs), including long genomic DNA, HMGB1, histones, intact or damaged mitochondria (potentially including mtDNA), full-length IL-33, IL-1α, ATP, and S100A9 (18, 19). Previous studies have found that elevated levels of MLKL mRNA in PBMCs of SLE patients are associated with renal damage and disease activity (20). Additionally, activation of necroptosis has been observed in patients with cutaneous vasculitis, ulcerative colitis, and psoriasis (21). In addition to the formation of necrosome mediated by RIPK1, RIPK3, and MLKL, necroptosis can also be induced through ZBP1, which senses viral infection in a RIPK3-dependent pathway, leading to necroptosis (22). This study found that necroptosis-related genes were enriched in PBMCs of SjD. Meanwhile, the level of HMGB1 in peripheral blood increased and was correlated with ESSDAI, suggesting that necroptosis is involved in the pathogenesis of SjD. Targeting necroptosis or HMGB1 could become new therapeutic targets for SjD.

## Subjects and methods

### Bioinformatics analysis of the GEO database

The mRNA expression profile dataset of GSE48378 was downloaded from the GEO^1^ database. GSE48378, which included transcriptomes of PBMCs from 11 patients with SjD and 16 HCs, was analyzed on the GPL5175 platform (Affymetrix human exon 1.0 ST array).

### Analysis of differential genes

The normalized expression matrix and the annotation files were downloaded from the GSE48378 database. The principal component analysis (PCA) was conducted. Because the fold change between the two groups is relatively small, and considering the presence of technical noise, we have set a relatively loose but reasonable threshold, that is, |log₂FC| >0.15, and adjusted *p*-value <0.05. Those that meet these two conditions are defined as differentially expressed genes. Then, GO and KEGG pathway enrichment were analyzed using the R and “Go plot” packages. The volcano plot was generated using the “ggplot2” package of R software. The heat map and box plot were generated using the “heatmap” and “ggplot2” packages of R software, respectively.

### Analysis of necroptosis-related genes

The correlations of the differentially expressed necroptosis genes were addressed using Spearman correlation in the R “corrplot” package. Then, the STRING database^2^ and Cytoscape software (version 3.7.3) were used to perform a PPI analysis of the differentially expressed necroptosis genes.

### Patients with SjD and healthy controls

A total of 20 patients newly diagnosed with SjD in Wuhan Tongji Hospital from 2024 to 2025 were enrolled, whereas patients combined with other autoimmune diseases or infections were excluded. Ten age- and sex-matched healthy controls (HCs) were recruited in the physical examination center of Wuhan Tongji Hospital. All the patients with SjD fulfilled the 2016 classification criteria proposed by the American College of Rheumatology (ACR)/European League Against Rheumatism (EULAR) group. This study was approved by the ethics committee of Tongji Hospital and all participants provided written informed consent.

### Isolation of human PBMC

Six-milliliter blood samples were collected in an ethylenediamine tetraacetic acid (EDTA)-K2 tube (BD Vacutainer) from all the participants. The PBMC was isolated by Ficoll (TBD Science, Tianjin, China) gradient centrifugation for 30 min at 700 G. The buffy coat was transferred to a new tube and subjected to phosphate buffer solution (PBS) washing two times. The final PBMCs were then transferred into 1 mL Trizol Reagent (Life Invitrogen, Carlsbad, CA, United States) in RNase-free tubes and stored at −80 °C until use.

### RNA extraction and RT-PCR

Total RNAs were isolated from the PBMCs and quantified. cDNAs were reverse transcribed from 500 ng total mRNA using a commercial cDNA Reverse Transcription Kit (Yeasen Biotechnology, Shanghai, China). MLKL, RIPK3, ZBP1, IRF9, CHMP1B, NLRP3, EIF2AK2, TLR4, STAT3, TNFRSF10B, and HMGB1 were determined by quantitative real-time PCR using SYBR Green reagents. Primers sequences utilized in the current study were as follows: MLKL forward: 5′-AGGAGGCTAATGGGGAGATAGA-3′, reverse: 5′- TGGCTTGCTGTTAGAAACCTG-3′; RIPK3 forward: 5′-ATGTCGTGCGTCAAGTTATGG-3′, reverse: 5′-CGTAGCCCCACTTCCTATGTTG-3′; ZBP1 forward: 5′-AACATGCAGCTACAATTCCAGA-3′, reverse: 5′-AGTCTCGGTTCACATCTTTTGC-3′; IRF9 forward: 5′-GCCCTACAAGGTGTATCAGTTG-3′, reverse: 5′-TGCTGTCGCTTTGATGGTACT-3′; CHMP1B forward: 5′-AAAGAACTGAGTAGGAGTGCCA-3′, reverse: 5′-TGTATCCTCGCAACTTCCATGT-3′; NLRP3 forward: 5′-GATCTTCGCTGCGATCAACAG-3′, reverse: 5′- CGTGCATTATCTGAACCCCAC-3′; EIF2AK2 forward: 5′- GCCGCTAAACTTGCATATCTTCA-3′, reverse: 5′-TCACACGTAGTAGCAAAAGAACC-3′; TLR4 forward: 5′-AGACCTGTCCCTGAACCCTAT-3′, reverse: 5′-CGATGGACTTCTAAACCAGCCA-3′; STAT3 forward: 5′- CAGCAGCTTGACACACGGTA-3′, reverse: 5′-AAACACCAAAGTGGCATGTGA-3′; TNFRSF10B forward: 5′- GCCCCACAACAAAAGAGGTC-3′, reverse: 5′-AGGTCATTCCAGTGAGTGCTA-3′; HMGB1 forward: 5′-TATGGCAAAAGCGGACAAGG-3′, reverse: 5′-CTTCGCAACATCACCAATGGA-3′; ß-Actin forward: 5’-CATGTACGTTGCTATCCAGGC-3′ and reverse: 5’-CTCCTTAATGTCACGCACGAT-3′.

### ELISA

To collect serum samples from SjD and healthy controls, follow the procedure below: Obtain informed consent from the participants and ensure proper ethical considerations are met. Collect blood samples from SjD and HCs using standard phlebotomy techniques. Centrifuge the blood samples at a suitable speed and duration to separate the serum from cellular components. Carefully transfer the serum into labeled cryovials. Store the cryovials at −80 °C until further use to maintain the stability of the samples. When ready to analyze the HMGB1 concentration, thaw the serum samples on ice. Follow the instructions provided by the manufacturer of the ELISA kit (CUSABIO, catalog number CSB-E08223h) to measure the HMGB1 concentration in human serum using the ELISA assay. Perform the ELISA assay according to the protocol provided by the manufacturer. Record and analyze the HMGB1 concentrations in the serum samples from SjD and HCs.

### Electron microscopy

Centrifuge to pellet the cells, discard the culture medium, and add electron microscopy fixative (Servicebio, G1102) for fixation at room temperature for 2 h. Then transfer the sample to 4 °C for storage. After washing, post-fix the sample with 2% osmium tetroxide solution under light-proof conditions for 2 h. Perform gradient dehydration using ethanol, followed by infiltration and embedding with epoxy resin. Section the samples, perform electron staining, and finally observe and acquire images via transmission electron microscopy (TEM).

### Statistical analysis

The statistical analyses were performed using R software (version 3.6.1) and GraphPad Prism software (version 7.0). Gene expression data of clinical samples were presented as mean ± standard error of the mean (SEM) and analyzed using an unpaired *t*-test. For normally distributed continuous variables, the unpaired *t*-test was used for analysis. For rates, the chi-square test or Fisher’s exact test was used. *p*-values less than 0.05 were considered statistically significant.

## Results

### Differentially expressed genes in the GSE48378 database

**Figure 1:**
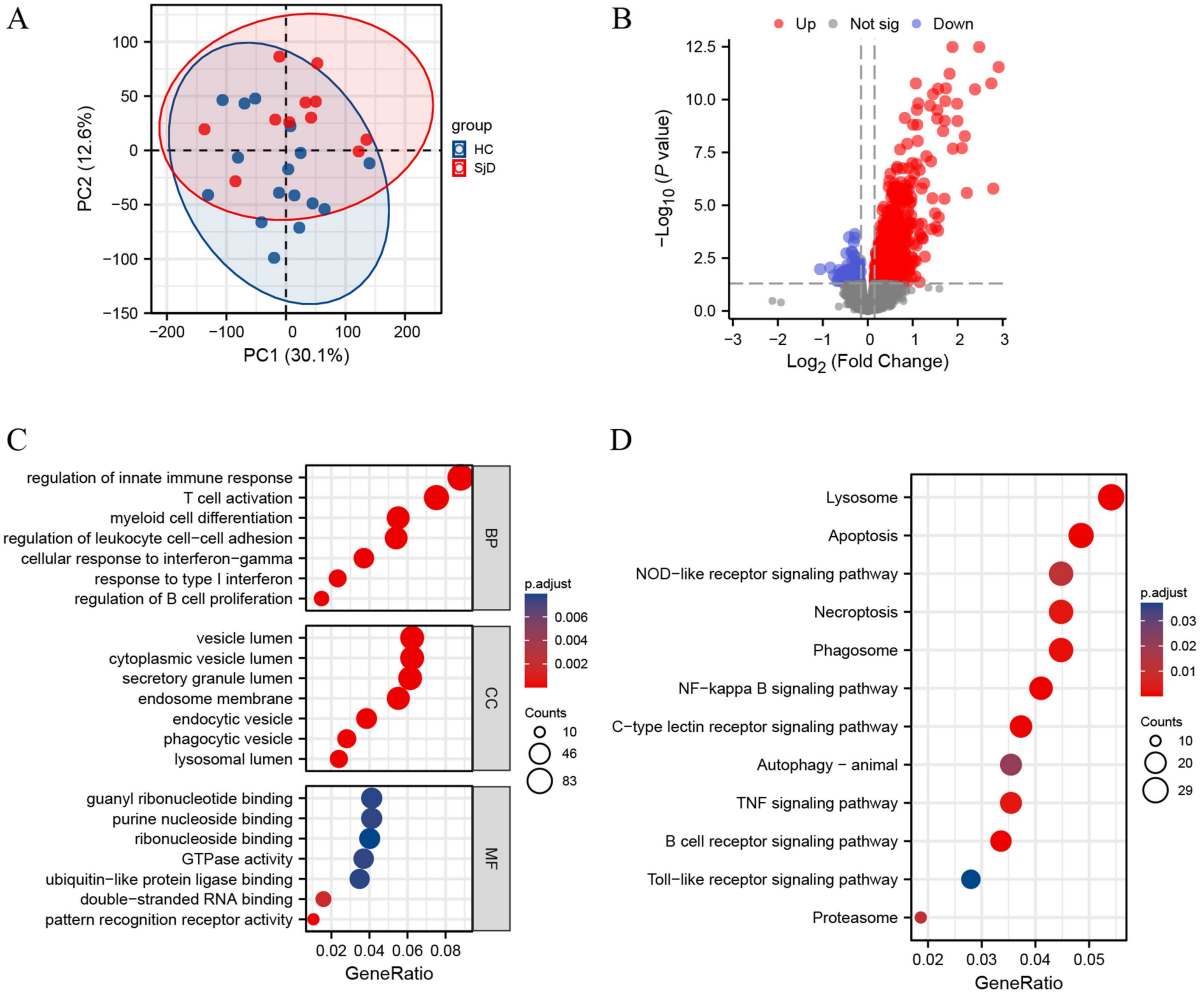
Differentially expressed genes in the GSE48378 database. **(A)** Principal component analysis for GSE48378. **(B)** Volcano plot of 1,159 differentially expressed genes. The red dots represent the significantly up-regulated genes, and the blue dots indicate the significantly down-regulated genes. **(C)** Gene Ontology (GO) enrichment analysis of differentially expressed genes. **(D)** The abnormal biological processes, especially different forms of cell death, are involved in the PBMCs of patients with SjD. BP, biological process; CC, cellular component; MF, molecular function.

PCA was conducted using the GSE48378 database from PubMed to evaluate the repeatability of the data. We found that the data repeatability was acceptable (Figure 1A). To obtain as many differentially expressed genes as possible, an adjusted *p*-value <0.05 was adopted as statistical significance. As shown in the volcano plot, we found that compared with the healthy controls (HCs), there were 1,146 differentially expressed genes in the SjD group, of which 1,029 were up-regulated, whereas 117 were down-regulated (Figure 1B). The GO and KEGG enrichment analyses were carried out to further address the potential biological pathways. The results displayed that the differentially expressed genes were mainly associated with biological processes of the regulation of internal immune response, T cell activation, myeloid cell differentiation, regulation of leukocyte cell adhesion, cellular response to interference gamma, response to type I interference, and regulation of B cell promotion. In addition, genes in the PBMCs of SjD associated with cell component (CC) (e.g., vesicle lumen, cytoplasmic vesicle lumen, secretory granule lumen, endosome membrane, endocytic vesicle, phagocytic vesicle, lysosomal lumen) and molecular functions (MF) (e.g., guanyl ribonucleotide binding, purine nucleotide binding, ribonucleoside binding, GTPase activity, ubiquitin-like protein ligase binding, double−stranded RNA binding, pattern recognition receptor activity) also significantly differed from those in HCs (Figure 1C).

In addition, different forms of cell death are involved in the SjD, including apoptosis, necroptosis, and autophagy. The apoptosis is considered to be non-inflammatory, and endogenous DAMPs are modified and silenced by apoptosis-related enzymes, but insufficient apoptosis clearance is considered to result in secondary necrosis. Autophagy is a process in which cells degrade non-essential or dysfunctional components into basic nutrients for absorption by lysosomes. Autophagy is generally regarded as a major anti-inflammatory process, but in certain circumstances, it can also promote inflammation. Compared with apoptosis and autophagy, necroptosis, as a main form of programmed necrosis, can be accompanied by significant inflammation. This study specifically focuses on necroptosis (Figure 1D).

### The necroptosis genes in SjD

**Figure 2:**
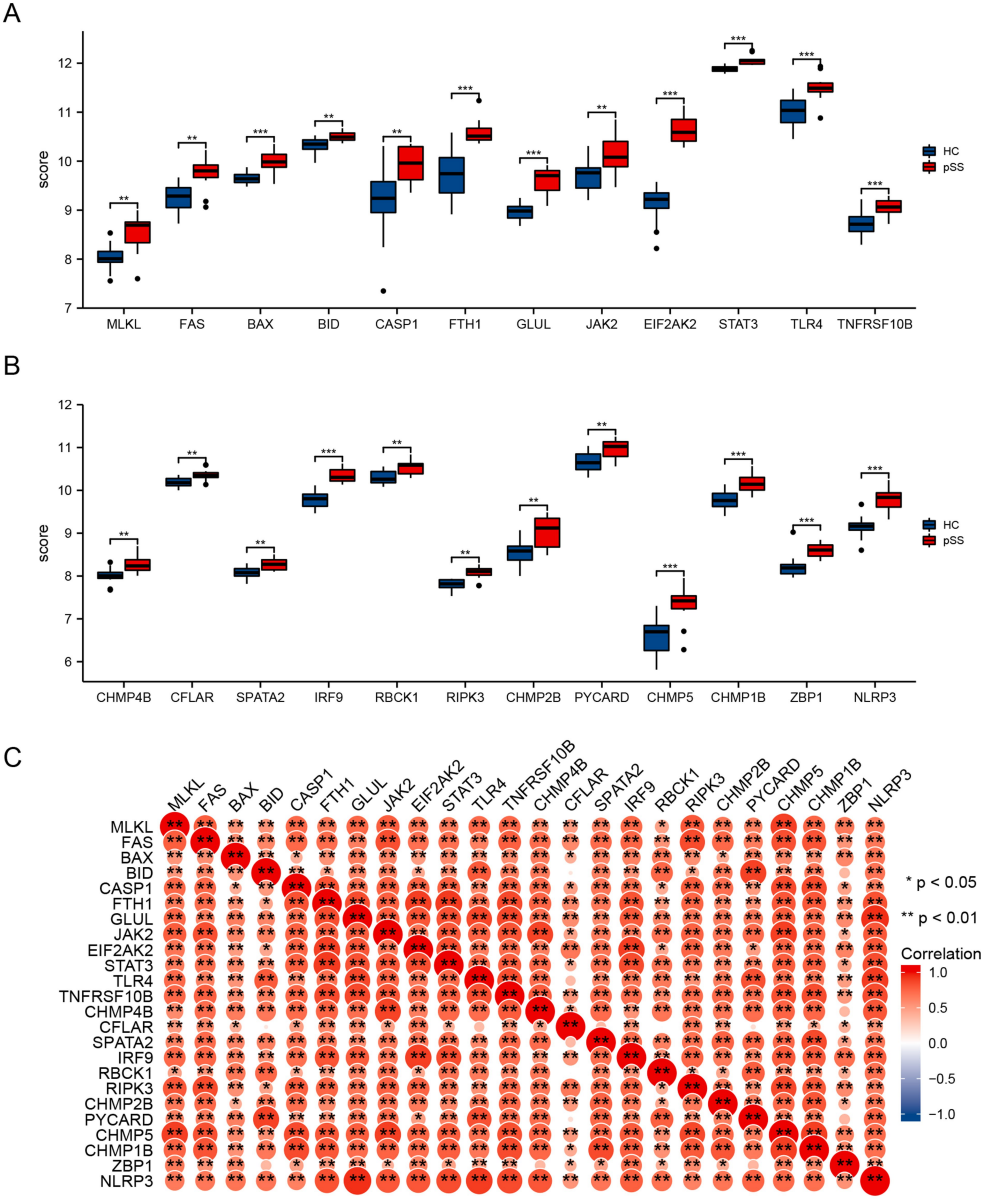
The differentially expressed necroptosis genes in the PBMCs of SjD and healthy controls. **(A,B)** The expression levels of the 24 differential genes associated with necroptosis in the PBMCs from 11 SjD and 16 healthy controls of the GSE48378 database. **(C)** Spearman correlation analysis of the 24 differentially expressed necroptosis-related genes.

We next determined the expression of differentially expressed genes associated with necroptosis in the PBMCs from 11 SjD and 16 healthy controls from GSE48378. All 24 necroptosis genes were up-regulated in the PBMCs of SjD, including MLKL, FAS, BAX, BID, CASP1, FTH1, GLUL, JAK2, EIF2AK2, STAT3, TLR4, TNFRSF10B, CHMP4B, CFLAR, SPATA2, IRF9, RBCK1, RIPK3, CHMP2B, PYCARD, CHMP5, CHMP1B, ZBP1, and NLRP3 (Figures 2A,B). A correlation analysis was conducted to further explore the correlations among the necroptosis genes. The results showed significant correlations among the 24 necroptosis-related genes, with only a few genes displaying no significant correlation (e.g., BID and CFLAR) (Figure 2C).

### PPI network analysis of the 24 up-regulated necroptosis genes in SjD

Then, a PPI network analysis was conducted to investigate the interactions among the enhanced 24 necroptosis genes in SjD. As expected, we found that the 24 necroptosis genes closely interacted with each other (Supplementary Figure 1A). CFLAR, STAT3, RIPK3, TLR4, MLKL, and CASP1 could interact with more than 10 necroptosis genes (Supplementary Figure 1B). Additionally, the expression profile of the up-regulated 24 necroptosis genes in the PBMCs in the participants of GSE48378 was also presented in a heat map (Supplementary Figure 1C).

### Verification of differentially expressed necroptosis genes using SjD samples

We recruited 10 HCs and 20 patients who were newly diagnosed with SjD. The median age of the HCs was 54 years with an interquartile range (IQR) of 18.25, while the median age of the SjD was 56.5 years with an IQR of 20. There was no statistically significant difference between the two groups. The percentage of females in HCs was 70%, while in SjD was 90%. However, there was no statistically significant difference. In terms of autoantibody presence, all HCs tested negative for autoantibodies. In SjD, the positive rate for antinuclear antibody (ANA) was 100%, Sjögren’s syndrome antigen A (SSA) was 75%, Sjögren’s syndrome antigen B (SSB) was 55%, and Ro52 was 75%. These findings showed a significant difference between the two groups. We further analyzed the EULAR Sjögren’s syndrome disease activity index (ESSDAI), erythrocyte sedimentation rate (ESR), immunoglobulin G (IgG), rheumatoid factor (RF), and the ratio of T helper cells (Th) to T suppressor cells (Ts) in the SjD group. The median and range of these parameters were as follows: ESSDAI: median 2.00, range (1.00, 6.00), ESR: median 14.00 mm/h, range (5.00, 56.00 mm/h), IgG: median 14.10 g/L, range (4.70, 27.80 g/L), RF: median 11.00 IU/mL, range (2.00, 207.00 IU/mL), Th/Ts ratio: median 1.545, range (0.59, 3.35), Focus score: median 1.00, range (1.00, 5.00). As for HCs, data regarding ESSDAI, ESR, IgG, RF, Th/Ts ratio, and focus score were not available (Table 1).

**Table 1:** Demographic and clinical characteristics of SjD at baseline.

Variables	Groups		*p-*value
	HCs	SjD	
Individuals/patients (*n*)	10.00	20.00	—
Age (years), median (IQR)	54.00 (18.25)	56.50 (20.00)	0.263
Female, *n* (%)	7.00 (70)	18.00 (90)	0.200
ANA, *n* (%)	0 (0)	20.00 (100.00)	<0.0001
SSA, *n* (%)	0 (0)	15.00 (75.00)	<0.001
SSB, *n* (%)	0 (0)	11.00 (55.00)	0.004
Ro52, *n* (%)	0 (0)	15.00 (75.00)	<0.001
ESSDAI, median (range)	NA	2.00 (1.00, 6.00)	—
ESR, median (range)	NA	14.00 (5.00, 56.00)	—
IgG, median (range)	NA	14.10 (4.70, 27.80)	—
RF, median (range)	NA	11.00 (2.00, 207.00)	—
Th/Ts, median (range)	NA	1.545 (0.59, 3.35)	—
Focus score, median (range)	NA	1.00 (1.00, 5.00)	—

HCs, healthy controls; SjD, Sjögren disease; IQR, interquartile range; ANA, antinuclear antibody; SSA, Sjögren’s syndrome antigen A; SSA, Sjögren’s syndrome antigen B; ESSDAI, EULAR Sjögren’s syndrome disease activity index; ESR, erythrocyte sedimentation rate; IgG, immunoglobulin G; RF, rheumatoid factor; Th, T helper cells; Ts, T suppressor cell.

**Figure 3:**
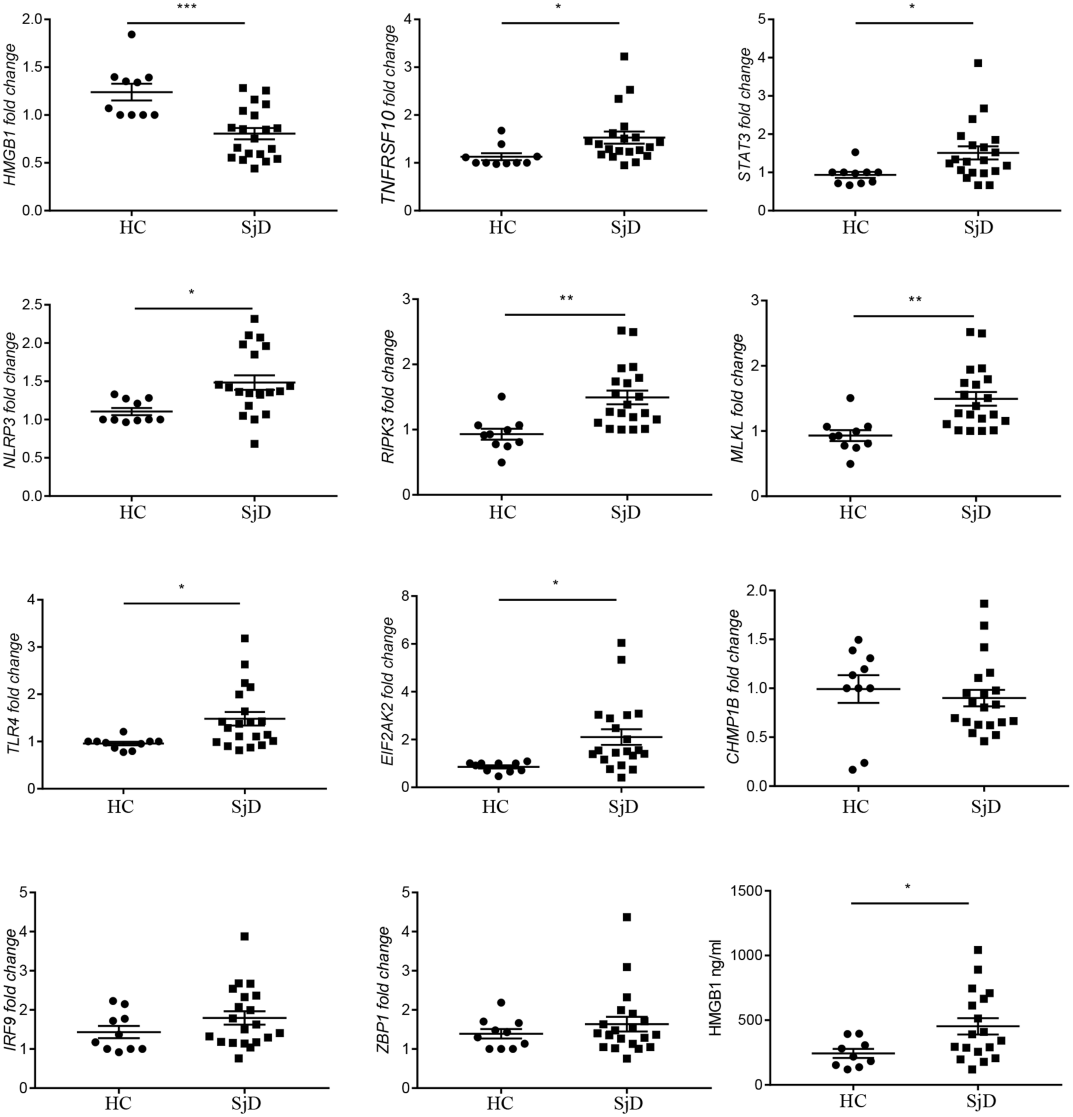
Verification of 10 differentially expressed necroptosis-related genes and comparing serum HMGB1 levels in SjD and healthy controls. The transcription levels of *HMGB1*, *TNFRSF10*, *STAT3*, *NLRP3*, *RIPK3*, *MLKL*, *TLR4*, *EIF2AK2*, *CHMP1B*, *IRF9*, and *ZBP1* in the PBMCs from 20 patients with SjD and 10 healthy controls using quantitative PCR. Serum HMGB1 levels were measured by ELISA. The data were presented as mean ± SEM. ^*^p < 0.05, ^**^p < 0.01, and ^***^p < 0.001.

**Figure 4:**
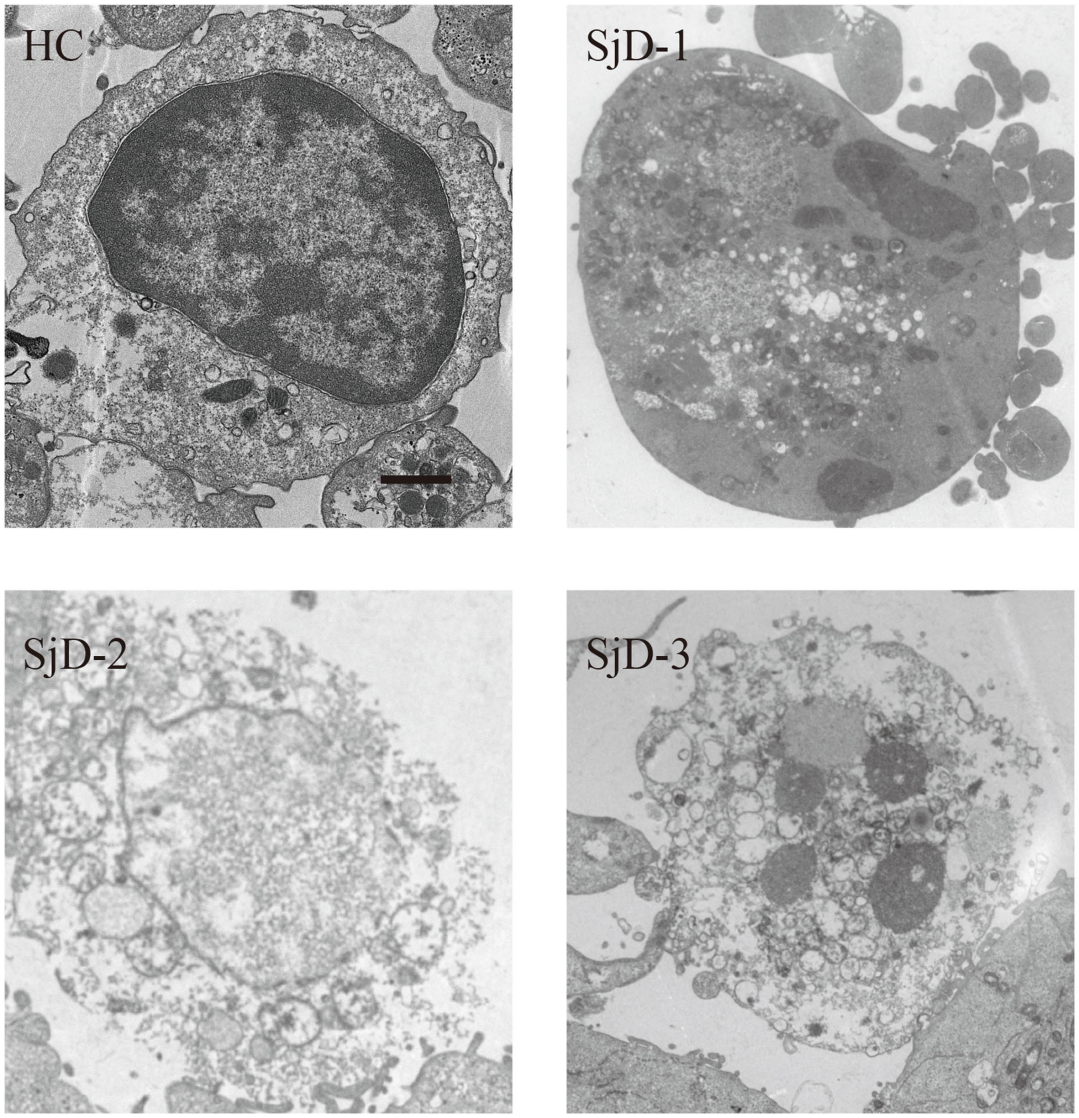
Electron microscopy revealed that there was necroptosis in the PBMC of SjD. We found that the typical manifestations of necroptosis were present in the PBMCs of SjD by electron microscopy, while this phenomenon was not observed in the healthy controls. The scale bar is 1 μm.

The transcription levels of the top eight differential necroptosis genes, two critical genes involved in necroptosis, and HMGB1 have been detected in the PBMCs from all participants. The quantitative PCR data demonstrated that the transcription levels of TNFRSF10, STAT3, NLRP3, RIPK3, MLKL, TLR4, and EIF2AK2 were significantly elevated in the PBMCs of SjD compared with those of HCs. However, HMGB1 transcription levels in SjD were significantly decreased. We observed that 50% of patients with SjD experienced lymphocyte reduction, suggesting that some activated lymphocytes (potentially high HMGB1 transcription levels) may undergo inflammatory cell death or further migrate to local tissues. To further verify the function, we measured HMGB1 concentration in serum. We removed two outliers from the SjD (2819.536 ng/mL and 1402.092 ng/mL) and excluded one case from the HCs because the data were below the detection limit. We found that serum HMGB1 levels were significantly increased in SjD (Figure 3), further suggesting HMGB1 release. To clarify that the PBMCs have undergone necroptosis and are involved in the release of HMGB1. Using electron microscopy, we observed necroptosis-like features in the PBMCs of the SjD. Still, the phenomenon was not observed in the HCs (Figure 4). Further analysis revealed that, in the selected layers, approximately 65% of patients with SjD had necroptotic-like cells. Necroptosis may be one of the reasons for the elevation of HMGB1 in peripheral blood. HMGB1 exhibits immunostimulatory properties and can act as an endogenous adjuvant. Numerous studies have revealed the role of HMGB1 in immune-related diseases. These findings suggest that PBMCs in SjD undergo necroptosis and may release HMGB1, which may contribute to the progression of SjD.

### Correlation analysis of MLKL and HMGB1 with clinical parameters of SjD

**Figure 5:**
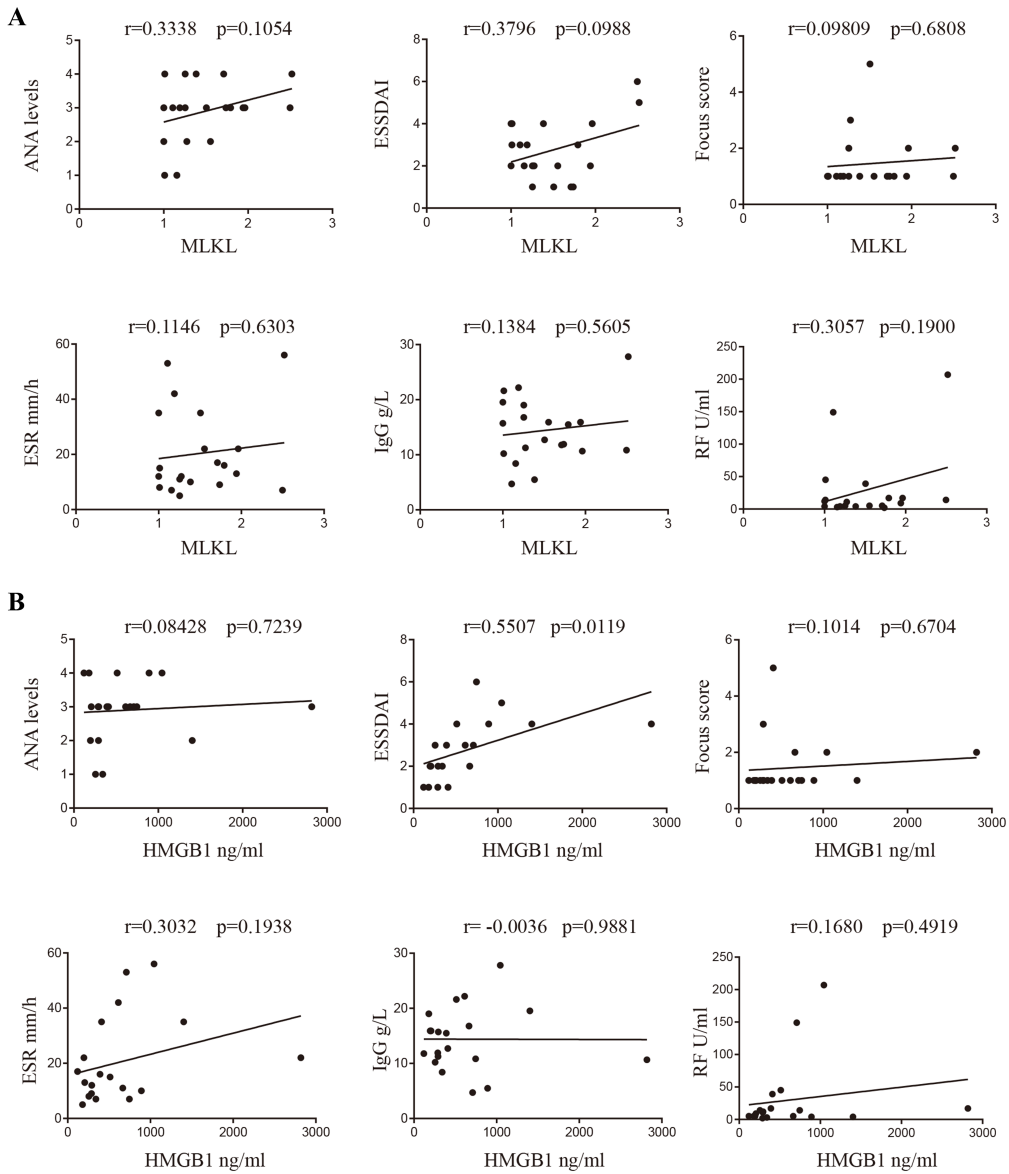
Correlation analysis of MLKL or HMGB1 with clinical parameters of SjD. **(A)** The correlations between MLKL and the clinical parameters (including ANA, ESSDAI, focus score of labial glands, ESR, IgG, and RF levels). **(B)** Similarly, the correlations between HMGB1 and clinical parameters (including ANA, ESSDAI, focus score of labial glands, ESR, IgG, and RF levels). Correlation coefficients and *p*-values are shown above the corresponding images.

The vital clinical data of patients with SjD were collected, including ANA (ANA, 1:100 was defined as level 1; 1:320 was defined as level 2; 1:1000 was defined as level 3; 1:3200 was defined as level 4), ESSDAI, focus score, ESR, IgG, and RF levels. We conducted a correlation analysis between the transcription levels of MLKL in PBMCs and the serum concentration of HMGB1 with the above-mentioned clinical parameters in SjD. The correlation coefficients (*p*-values) between MLKL and ANA, ESSDAI, ESR, focus score, RF, and IgG levels were as follows: MLKL and ANA: *r* = 0.3338 (*p* = 0.1054), MLKL and ESSDAI: *r* = 0.3796 (*p* = 0.0988), MLKL and ESR: *r* = 0.09809 (*p* = 0.6808), MLKL and focus score: *r* = 0.1146 (*p* = 0.6303), MLKL and RF: *r* = 0.1384 (*p* = 0.5605), MLKL and IgG levels: r = 0.3057 (*p* = 0.1900). The correlation coefficients (*p*-values) between HMGB1 and ANA, ESSDAI, ESR, focus score, RF, and IgG levels were as follows: HMGB1 and ANA: *r* = 0.08428 (*p* = 0.7239), HMGB1 and ESSDAI: *r* = 0.5507 (*p* = 0.0119), HMGB1 and ESR: *r* = 0.1014 (*p* = 0.6704), HMGB1 and focus score: *r* = 0.3032 (*p* = 0.1938), HMGB1 and RF: *r* = −0.0036 (*p* = 0.9881), HMGB1 and IgG levels: *r* = 0.1680 (*p* = 0.4919) (Figure 5). We find HMGB1 is positively correlated with ESSDAI, which may represent a novel therapeutic target.

## Discussion

SjD is the second most common rheumatic disease, following rheumatoid arthritis. It is characterized by dysfunction of the exocrine glands and presents with dry mouth and dry eyes, and can also involve multiple systems and even lead to lymphoma (23). Although there has been a deeper understanding of the pathogenesis of SjD, including the interaction between salivary gland epithelial cells and immune cells, interactions among immune cells, involvement of the extracellular matrix, viral infections, and genetic and gender factors, the specific mechanisms are still unclear. Furthermore, SjD is a highly heterogeneous and complex disease, which adds to the difficulty of treatment (9, 10, 24). Although some clinical studies have shown promising results for the treatment of SjD, including hydroxychloroquine combined with leflunomide (25), ianalumab (VAY736) (26), low-dose IL-2 (27), baricitinib (28), and telitacicept (29), the treatment options for SjD are still relatively limited compared to other rheumatic diseases, such as rheumatoid arthritis. Therefore, there is still an urgent need for further research on the treatment of SjD. Cell death encompasses various forms, including apoptosis, necrosis, pyroptosis, necroptosis, PANoptosis, ferroptosis, cuproptosis, autophagy, and ENTosis. These different forms of cell death play extensive roles in the pathophysiology of diseases (17, 30). Among them, necroptosis is a programmed necrosis pathway, which depends on activating RIPK1, RIPK3, and MLKL, leading to cell swelling, membrane rupture, and release of cytoplasmic contents. The classical process of necroptosis involves the binding of TNF to TNFR1, which induces the formation of complex I, including RIPK1, TRADD, cIAP1, and cIAP2. When RIPK1 is not ubiquitinated, and the activity of caspase-8 is blocked, RIPK1 phosphorylates and activates RIPK3. Eventually, MLKL is phosphorylated, leading to cell lysis. Necroptosis is associated with significant inflammation and involves various biological processes such as immunity, inflammation, and tumors (31, 32). In this study, we discovered that 24 genes related to necroptosis were significantly upregulated in the PBMC of SjD compared to the control group through bioinformatics. This suggests that necroptosis may be involved in the pathogenesis of SjD. It is worth mentioning that among the 24 necroptosis-related genes we discovered, several well-known genes involved in inflammation were included, such as STAT3, TLR4, etc. In fact, inflammation is an important trigger for necroptosis, and these genes are involved in multiple biological processes. In a recent toxicological study, STAT3 activation was shown to play a critical role in patulin-induced necroptosis in renal cells (33). In addition, TLR4 has also been identified as an independent trigger of necroptosis (34).

To validate these, we collected peripheral blood samples from 10 HCs and 20 patients with SjD. We extracted PBMCs and performed transcriptional level validation for eight significantly different genes, as well as MLKL and RIPK3. Additionally, we examined the differences in HMGB1 expression between SjD and HCs. We observed elevated transcription levels of TNFRSF10, STAT3, NLRP3, RIPK3, MLKL, TLR4, and EIF2AK2 in SjD, consistent with bioinformatics. Although an upregulation of key necroptosis genes and related pathways was founded in our transcriptomic analysis, it may merely reflect a transcriptional program conducive to the initiation of necroptosis, as necroptosis primarily depends on post-translational modifications (such as phosphorylation) of key proteins—including RIPK1, RIPK3, and MLKL—as well as the formation of protein complexes, highlighting the necessity of verifying our results at the protein level in the future. Interestingly, we also found a decrease in the transcription level of HMGB1 in SjD. Therefore, we further evaluated HMGB1 levels in the serum of SjD, and we found higher HMGB1 levels in the serum of SjD. This seems to contradict the expected inflammatory response associated with necroptosis. But this is also logical. Firstly, activated lymphocytes (potentially high HMGB1 transcription levels) may undergo inflammatory cell death or further migrate to tissues. Secondly, high levels of HMGB1 in serum may, through an unclarified feedback mechanism, inhibit the transcription of HMGB1 in PBMCs. Lastly, continuous immune inflammation may lead to the exhaustion of lymphocytes and a decrease in the transcriptional activity of HMGB1. Previous studies have found that there is a significant amount of extracellular HMGB1 present around infiltrating mononuclear cells in the labial salivary gland tissue of SjD (35, 36). Additionally, another study has found higher levels of HMGB1 in the serum of SjD (37). These findings suggest a pro-inflammatory role for HMGB1 in SjD. Nevertheless, it is important to acknowledge that serum HMGB1 may originate from multiple cellular sources, including but not limited to circulating PBMCs, tissue-resident immune cells, or other damaged cells. In our study, the elevation of HMGB1 may therefore reflect a systemic inflammatory state. The necroptosis observed in PBMCs in the present study could represent only one possible contributing factor to the increased serum HMGB1 levels. Definitive confirmation of this causal relationship will require more direct experimental approaches, such as cell-specific HMGB1 knockout models, *in vitro* supernatant assays, or *in vivo* tracing techniques. In addition, using electron microscopy, we found typical morphological alterations in PBMCs from SjD patients consistent with necroptosis, which also may serve as a source of autoantigens and contribute to the development and progression of the disease. Considering the limitations of electron microscopy technology in identifying necrotic apoptosis, such as bias in morphological interpretation and inability to provide molecular level confirmation, our results mainly provide auxiliary evidence for the occurrence of necroptosis in PBMCs from SjD patients. Molecular biology methods such as detection of phosphorylated MLKL are still necessary in the future study.

Previous studies have also found that PBMCs undergo necroptosis in patients with SLE. In SLE patients, MLKL mRNA levels in PBMCs are significantly higher than in rheumatoid arthritis (RA) patients and HCs. Antinuclear antibody (ANA) positive SLE patients and renal involvement SLE patients had higher MLKL mRNA levels. Additionally, MLKL mRNA levels are positively correlated with C-reactive protein (CRP), ESR, and serum IgG levels (20). In addition to SLE, necroptosis also plays a role in the pathogenesis of rheumatoid arthritis (RA) and gout (38, 39). These findings suggest that necroptosis is involved in various autoimmune diseases by releasing DAMPs and providing potential antigens to promote disease progression. Therefore, targeting necroptosis could be a potential therapeutic approach. It should be noted that local events within the glands including necroptosis, are likely more critical in the pathogenesis of SjD; thus, the alterations we observed in PBMCs may reflect systemic immune dysregulation rather than serving as direct mediators of glandular damage. Fortunately, we also find a positive correlation between serum HMGB1 concentrations and ESSDAI in SjD. These results suggest the involvement of necroptosis in the development of SjD and highlight it as a potential therapeutic target. In fact, several necroptosis inhibitors, such as the RIPK1 inhibitors GSK-872 and necrostatin-1, have demonstrated efficacy in preclinical models of other autoimmune or inflammatory diseases (40). These agents could be prioritized for clinical and translational research in SjD. For instance, future studies may initially evaluate their safety and therapeutic effects on salivary gland inflammation and function in established animal models of SjD. Moreover, given the limited efficacy and inter-individual variability associated with current therapeutic options for SjD—including hydroxychloroquine and rituximab—necroptosis inhibitors may be considered for combination with existing anti-inflammatory or immunomodulatory agents to enhance therapeutic benefit or reduce adverse effects. Additionally, our finding that necroptosis-related molecular markers in PBMCs correlate with disease activity suggests that these molecules could serve as potential biomarkers for stratifying patients who may benefit from necroptosis-targeted therapies, thereby facilitating a more personalized treatment approach.

Indeed, there are limitations to our study. The sample size was relatively small, and it was conducted at a single center. Larger validation cohorts from multiple centers are necessary for future research. Additionally, it is important to note that our analysis is based on bulk RNA-seq data from PBMCs, which are a heterogeneous mixture of cell types. The observed upregulation of the 24-gene necroptosis signature in SjD could potentially be influenced by changes in the relative proportions of immune cells, such as an increase in monocytes, which may have a higher basal expression of certain necroptosis-related genes compared to lymphocytes. Given the heterogeneity of PBMCs, more studies and updated methods, such as single-cell RNA sequencing technology, are needed to identify which particular cell types in PBMCs are more prone to undergoing necroptosis and to elucidate the specific mechanisms involved. Moreover, due to the limited availability of samples, we did not assess phosphorylated MLKL. Future studies may need to evaluate the occurrence of necroptosis in salivary gland tissue.

Nevertheless, our study provides the first indication that PBMCs in SjD may undergo necroptosis and potentially contribute to disease progression through the release of HMGB1—a finding that warrants confirmation through more direct experimental approaches in future studies. Inhibiting the necroptosis in PBMCs of SjD may represent a new potential therapeutic target. These findings contribute to a better understanding of the complex pathogenesis of SjD.

## Data Availability

The raw data supporting the conclusions of this article will be made available by the authors, without undue reservation.

## Footnotes

1
https://www.ncbi.nlm.nih.gov/geo/

2
https://string-db.org/

## Glossary

SjD: Sjögren disease
GO: Gene Ontology
KEGG: Kyoto Encyclopedia of Genes and Genomes
HMGB1: High mobility group box 1
ELISA: Enzyme-linked immunosorbent assay
RT-PCR: Real-time polymerase chain reaction
PPI: Protein–protein interactions
PBMCs: Peripheral blood mononuclear cells
MLKL: Mixed lineage kinase domain-like
BAX: BCL2 associated X
BID: BH3 interacting domain
CASP1: Caspase1
FTH1: Ferritin heavy chain 1
GLUL: Glutamate-ammonia ligase
JAK2: Janus kinase 2
EIF2AK2: Eukaryotic translation initiation factor 2 alpha kinase 2
STAT3: Signal transducer and activator of transcription 3
TLR4: Toll-like receptor 4
TNFRSF10B: TNF receptor superfamily member 10b
CHMP4B: Charged multivesicular body protein 4B
CFLAR: CASP8 and FADD like apoptosis regulator
SPATA2: Spermatogenesis associated 2
IRF9: Interferon regulatory factor 9
RBCK1: RANBP2-type and C3HC4-type zinc finger containing 1
RIPK3: Receptor-interacting serine-threonine kinase 3
CHMP2B: Charged multivesicular body protein 2B
PYCARD: PYD and CARD domain containing
CHMP5: Charged multivesicular body protein 5
CHMP1B: Charged multivesicular body protein 1B
ZBP1: Z-DNA binding protein 1
NLRP3: NLR family pyrin domain containing 3
