# Correction: Identification and validation of necroptosis-related genes in peripheral blood mononuclear cells of Sjögren disease

**DOI:** 10.3389/fmed.2026.1849017

**Published:** 2026-04-22

**Authors:** Yuanji Dong, Lingli Dong, Rongfen Gao

**Affiliations:** 1 Department of Rheumatology and Immunology, Tongji Hospital, Tongji Medical College, Huazhong University of Science and Technology, Wuhan, Hubei, China; 2 Department of Rheumatology, The Second Affiliated Hospital of Zhejiang University School of Medicine, Hangzhou, Zhejiang, China; 3 Department of Rheumatology and Immunology, Tongji Medical College and State Key Laboratory for Diagnosis and Treatment of Severe Zoonotic Infectious Disease, Huazhong University of Science and Technology, Wuhan, Hubei, China

**Keywords:** HMGB1, necroptosis, PBMCs, Sjögren disease, therapeutic target

The order of authors in the author list of the published paper was erroneously given as:

Yuanji Dong, Rongfen Gao and Lingli Dong

The correct author list reads:

Yuanji Dong, Lingli Dong and Rongfen Gao

The author contributions statement was erroneously published as:

YD: Data curation, Formal analysis, Funding acquisition, Writing – original draft. RG: Data curation, Formal analysis, Writing – review & editing. LD: Funding acquisition, Project administration, Supervision, Writing – review & editing.

The correct author contributions statement is:

YD: Data curation, Formal analysis, Funding acquisition, Writing – original draft. LD: Funding acquisition, Writing – review & editing. RG: Data curation, Formal analysis, Supervision, Writing – review & editing.

Author Lingli Dong was erroneously assigned as corresponding author. The correct corresponding author is Rongfen Gao.

Authors Yuanji Dong and Rongfen Gao were erroneously assigned as equal contributing authors. The statement has been removed.

The original version of this article has been updated.

