# Supplementary material for: Identification and validation of necroptosis-related genes in peripheral blood mononuclear cells of Sjögren disease
Source: Front Med (Lausanne). 2026 Mar 26;13:1727374. doi: 10.3389/fmed.2026.1727374 (PMC13062312; doi:10.3389/fmed.2026.1727374)
Supplement: Supplementary file 1 [file Image_1.pdf]

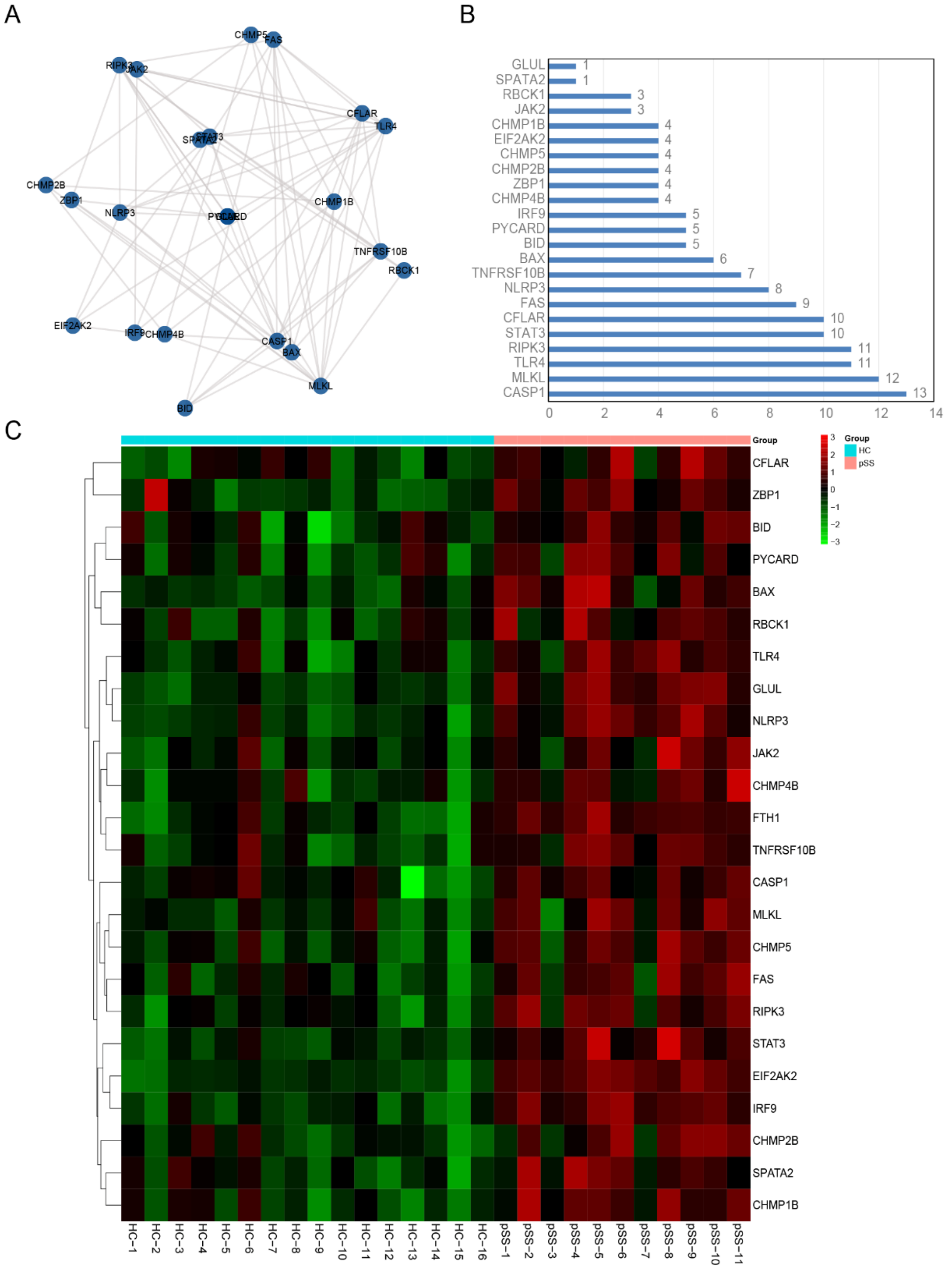

Supplementary figure 1: **PPI network analysis of the 24 differentially expressed necroptosis genes in the PBMCs of SjD and healthy controls**. (A) The PPI among 24 differentially expressed necroptosis-related genes. (B) The interaction number of each differentially expressed necroptosis-related gene. (C) Heatmap of the 24 differentially expressed necroptosis-related genes in SjD and healthy controls.
